# Improving the Quality of Dementia Care in General Practice: A Qualitative Study

**DOI:** 10.3389/fmed.2020.600586

**Published:** 2020-11-25

**Authors:** Meghan Bourque, Tony Foley

**Affiliations:** Department of General Practice, University College Cork, Cork, Ireland

**Keywords:** dementia, primary care, general practice, qualitative study, general practitioner (GP)

## Abstract

**Background:** General Practitioners (GPs) play a central role in caring for people with dementia. There is a growing demand for GP-led community-based dementia care, as advocated in the Irish National Dementia Strategy (INDS). However, there is a paucity of research exploring GPs' views on dementia care since publication of the INDS. The aim of this qualitative study is to develop a deeper understanding of how to improve the quality of dementia care in General Practice, explored from the perspective of Irish GPs.

**Methods:** Semi-structured interviews were conducted with GPs. GPs who completed the “Dementia in Primary Care” CPD module at University College Cork in Ireland were purposively recruited. Interviews were audio-recorded, transcribed, and analyzed by thematic analysis.

**Results:** 12 interviews were conducted with 7 female and 5 male participants. Experience in General Practice ranged from 3 to 32 years. Most GPs practiced in mixed urban-rural settings (*n* = 9) and had nursing home commitments (*n* = 8). The average interview length was 45 minutes. Six major themes emerged from the data set, including resourcing primary care, addressing disparities in secondary care, community-centered care as patient-centered care, linking a dementia network, universal access to care, and raising public awareness.

**Conclusion:** GPs find dementia care to be a complex and challenging aspect of primary care. While education and training is advocated by GPs, service delivery must be reconfigured. This will necessitate adequate financial resourcing and the restructuring of community-based dementia care services.

## Introduction

Dementia is a syndrome characterized by a progressive decline in cognitive function and behavior that interferes significantly with a person's ability to maintain their activities of daily living (1). As the disease progresses, people with dementia require a considerable amount of care from a range of multidisciplinary clinicians. General practitioners (GPs) are often the first healthcare professionals to be consulted when dementia is suspected by a patient or their family. The average GP diagnoses one or two new patients with dementia each year and manages 12 to 15 patients with dementia on an average list size (2). The Irish National Dementia Strategy (INDS), published in 2014, highlighted the pivotal role played by GPs in the care of patients with dementia and advocated community-based care (3). Other national dementia strategies across Europe have similarly emphasized the importance of the GP in caring for this patient cohort (4, 5). As the Irish population ages the prevalence of dementia will rise, with numbers expected to double by 2036 (6, 7). Hence, the demand for specialized dementia care in primary care will inevitably increase.

Over the past few decades various initiatives have been formulated to improve the quality of dementia care in Ireland. They have highlighted the need for a social model of dementia that is focused on care in the community and maintaining the “personhood” of the patient with dementia (8). A major focus of the INDS was to develop resources to educate GPs on the provision of dementia-specific care in the community. These educational resources aimed to address knowledge gaps highlighted by GPs in previous research (9, 10). One example of this is the development a dementia-specific training module for GPs, “Dementia in Primary Care” (DIPC), which was initiated at University College Cork in 2017.

It is timely to examine perspectives on the quality of dementia care in Ireland in order to evaluate whether the INDS has led to quality improvements in dementia care. Similar research has not been undertaken since the publication of the INDS. Further, Ireland's Health Service Executive (HSE) recently announced funding for structured programs for chronic disease management in primary care; however, dementia was not included in this program (11).

The aim of this study is to develop a deeper understanding of how to improve the quality of dementia care in General Practice as viewed from the perspective of GPs. The study objectives include the identification of facilitators and barriers to dementia care, to gain perspectives on different strategies to enhance the delivery of care, and to explore quality improvement initiatives in practice.

## Methods

### Study Design

A qualitative study involving semi-structured, face-to-face interviews was performed. A qualitative approach was selected to thoroughly explore the views and experiences of GPs on quality improvement of dementia care. This approach enables the researcher to investigate in-depth perspectives on complex issues and related factors, with the desired goal of making conceptual generalizations from the local context of study (12, 13).

### Sampling & Recruitment

GP participants of the first two iterations of the DIPC module (2017 and 2018) were purposively recruited for the study. The DIPC module aims to upskill GPs on the diagnosis and management of dementia. This cohort was chosen because of their dementia expertise and their particular interest in quality improvement in the field of dementia care.

Recruitment packages (Appendix 1 in Supplementary Material) were sent to GPs by post. Participants were asked to complete a participation form, which was then returned to the Department of General Practice at UCC. GPs who were interested in participating were subsequently contacted by telephone to schedule an interview. Each participant received an outline of the topic guide (Appendix 2 in Supplementary Material) in advance of the interview.

### Study Measures

A quantitative data collection sheet (Appendix 2 in Supplementary Material) and a qualitative interview topic guide (Appendix 3 in Supplementary Material) were used to gather relevant data from each participant. Both tools were developed based on the professional expertise of one of the authors (TF), who is a practicing GP with years of expertise in dementia care and qualitative research.

The data collection sheet (Appendix 2 in Supplementary Material) was drafted to collect demographic characteristics of GP participants. The research team consulted the literature for comparable qualitative research to ensure relevant details were included.

The topic guide (Appendix 3 in Supplementary Material) was iteratively developed through a process of consensus with the research team following review of relevant literature in the field. Modifications were made to the guide throughout the study. The topic guide was broken into three sections, including an overview of the participants' practice and approach to dementia care, their opinions on the quality of dementia care, and their experience auditing dementia care.

### Data Collection

The study was carried out across Ireland. Semi-structured, face-to-face interviews were conducted between February to December of 2019 at locations suitable to each participant. One researcher (MB), a final year graduate entry medical student, conducted all of the interviews. Written informed consent and the data collection sheet were completed prior to each interview. Field notes were made during and after the interview to record relevant non-verbal information observed. All interviews were audio-recorded and transcribed verbatim by the interviewer (MB). The transcripts were subsequently checked against the original audio recordings for accuracy. Transcripts were de-identified and assigned anonymized codes (ex. GP01) to protect participant identity. In addition, all identifiable information was removed from selected quotes to ensure confidentiality.

### Data Analysis

The principles of thematic analysis outlined by Braun and Clarke were employed to analyze the data set (14). The first four interviews were extensively reviewed and independently coded by the research team (MB, TF). The research team then met to examine the convergence and divergence of the preliminary coding and to discuss emerging themes. The rest of the data set was extensively reviewed and coded by a single researcher (MB). Analysis of the data took place concurrently with data collection, allowing for emerging themes to be explored in subsequent interviews. The research team met regularly to ensure a thorough and comprehensive coding process that supported the development of themes that accurately represented the data set. A thematic map of the data set (Appendix 4 in Supplementary Material) was reviewed and finalized before interpretive analysis commenced. NVivo 12 qualitative data software was used for data analysis and management. The authors adhered to the consolidated criteria for reporting qualitative research (COREQ) statement in reporting the findings of the study (15).

## Results

Twelve interviews were conducted in total. Overall, conceptual data saturation was reached after 8 interviews. Four more interviews were subsequently conducted, confirming data saturation (16). This is in accordance with the stopping criterion for data saturation, which outlines that saturation is achieved after three interviews with no new shared beliefs (16). Interviews were, on average, 45 minutes in duration (range 29–60 min).

Participant characteristics were varied, as shown in Table 1. Seven female and five male GPs participated in the study. The majority of participants ranged in age from 40 to 59 years (*n* = 10). The average length of practice experience was 17.2 years (range 3–32 years). Most GPs worked in a mixed urban-rural and group practice setting (≥2 GPs). More than half of the GPs held nursing home commitments.

**Table 1:** Participant demographics.

**ID**	**Sex**	**Age**	**Years qualified**	**Practice setting**	**Practice type (# GPs)**	**Total patients**	**Dementia patients (% total)**	**Nursing home commitments?**	**Interview duration (min)**
*GP01*	M	40–49	16	Mixed	4	7,000	19 (0.27%)	No	58:04
*GP02*	F	40–49	7	Rural	2	1,300	20 (1.54%)	Yes	48:24
*GP03*	M	40–49	10	Mixed	4	5,000	40 (0.8%)	Yes	59:47
*GP04*	F	30–39	3	Mixed	7	7,000	50 (0.71%)	Yes	42:22
*GP05*	F	40–49	18	Mixed	4	4,500	22 (0.49%)	No	47:33
*GP06*	F	50–59	21	Rural	4	4,500	30 (0.67%)	Yes	48:53
*GP07*	M	60–69	32	Mixed	4	8,000	46 (0.58%)	Yes	44:41
*GP08*	F	50–59	25	Mixed	9	15,000	86 (0.57%)	Yes	28:38
*GP09*	M	50–59	30	Mixed	5	6,000	50 (0.83%)	No	39:11
*GP10*	M	40–49	10	Mixed	3	3,000	20 (0.67%)	Yes	36:52
*GP11*	F	50–59	25	Urban	1	2,000	75 (3.75%)	No	31:49
*GP12*	F	40–49	9	Mixed	4	3,000	60 (2%)	Yes	50:37

### The Role of the GP

#### Challenges of Care

GPs described the intensity and demands of caring for patients with dementia. While numbers of consultations with patients with dementia were relatively small, participants reported a mismatch between the number of consultations and the workload involved in dementia care in General Practice.

“*I don't think it's a big slice of the actual pie of care we take up. But if I had to draw up a pie of problem care then it would be a much bigger chunk […] How much effort it takes, how much mental energy it takes, is way out of proportion to the amount of work you're doing.” (GP09)*

Regarding the timely diagnosis of dementia, GPs described their pivotal role in recognizing early signs but also explained the challenges of time and clinic complexity.

“*A lot of times our diagnosis is evolving. They are coming in maybe at 65 and there are a few questions about something and then it evolves over the next few years when it becomes very obvious.” (GP01)*

“*It is very hard to pick it up in a 10 to 15 minute consultation […] So it's generally through the patient's own concerns or the family's concerns that we pick it up.” (GP09)*

#### Referral to Secondary Care

While participants described their role in recognizing symptoms, confirmation of the diagnosis of dementia was deemed to be the role of a secondary care specialist.

“*The diagnosis is made with the subtype by the geriatrician so it's very different to say cardiovascular disease or diabetes […] where the GP can make the diagnosis and make a management plan and then implement that management plan.” (GP10)*

#### Behavioral and Psychological Symptoms of Dementia

Following a formal diagnosis of dementia, GPs reported that their role was initially *ad hoc* and reactive. As the patient's clinical condition deteriorates they required more frequent, intensive care and follow-up.

“*We do not tend to [schedule regular appointments] unless there is another medical problem or unless there is a legal issue or they need a prescription change or renewal.” (GP02)*

“*Once they start developing behavioral problems […] then they start attending quite frequently. Behavioral problems or sleep disturbance or falls – those would be the triggers that would cause people with dementia to start attending more frequently.” (GP02)*

#### Nursing Home Care

The majority of GPs interviewed provided care in nursing homes. Participants confirmed that dementia care took up a much larger portion of nursing home care relative to care in the community. Participants explained that the care provided to patients with dementia in nursing homes is more frequent (daily to quarterly) and structured relative to community-based care.

“*In the nursing home unit we go down pretty much daily between the four of us and we are on call if there is an issue.” (GP03)*

“*At the nursing homes most patients end up being seen quite frequently because we do reviews on them […] We would often review their medications and see what we could cut out. They are seen at least every three months if we are not called to them beforehand.” (GP04)*

Participants described the complex care needs of patients with dementia in nursing homes, particularly those in more advanced stages of the disease. Their care efforts focused on advanced care planning and deprescribing, which is often met with resistance.

“*We're pretty good in the nursing homes in that most of our patients now would either have [advanced directives] decided or they are in the process of letting the family think about it.” (GP04)*

“*Deprescribing is very hard to do in nursing homes because [staff and families] don't want you to deprescribe. Nursing homes are barriers to deprescribing that's for sure.” (GP08)*

### Improving Dementia Care

GPs suggested a number of ways to overcome barriers and improve the quality of dementia care in General Practice. Six main themes emerged regarding quality improvement strategies.

1. Resourcing Primary Care
2. Addressing Disparities in Secondary Care
3. Community-Centered Care Equals Patient-Centered Care
4. Linking A Dementia Network
5. Universal Access to Care
6. Raising Public Awareness.

### Theme 1: Resourcing Primary Care

#### GP Remuneration & Structured Care

The resourcing of structured care for chronic diseases was deemed to be wholly inadequate by participants. While chronic disease management programs have recently been initiated in Irish General Practice for four chronic diseases (diabetes, asthma, chronic obstructive pulmonary disease and heart disease), dementia has not been included in this program (11). This was regarded as a major obstacle to comprehensive care for patients with dementia.

“*We get paid just for acute care of these people. We have for some reason taken on holistic, comprehensive care but our contract with the government for managing these people is for acute care, referring them on for secondary care and repeat prescriptions.” (GP01)*

GPs described the need for dementia to be recognized and included in a chronic disease management program in order to facilitate planned care and to afford enough time for long, complex consultations.

“*It's very difficult in a ten-minute consultation to do a proper assessment when you're in the early stages. I suppose often times we end up seeing them and bringing them back and maybe bringing them back again and having to divide it up […] Even doing the mental test score on them it all takes time and it's very difficult to fit that into the working day.” (GP06)*

One GP reported their experience applying an informal structured approach for one of their patients with dementia.

“*I have one lady now it just worked beautifully. I was able to bring her back on a regular basis and it worked very well. I'd sort of say, ‘Look it, we'll see you in three or four weeks and we'll cut this down another bit.’ (GP05)*

The benefit of a structured approach was further emphasized by participants' feedback on creating a dementia register and subsequently performing a dementia-focused audit as part of the DIPC module.

“*All of our dementia patients are flagged […] Now when you click on their file it comes up in the problem box in the corner so it is really clear straight away for everybody.” (GP04)*

However, this viewpoint was not unanimous. One GP argued against the value of regular scheduled visits for patients with dementia.

“*Yeah I could see them every three months but I'm not sure what for. And you might pick up some deterioration a little bit earlier but that's 15 minutes to half-an-hour gone for very little to show for it.” (GP10)*

Participants emphasized that improvement in the quality of dementia care will not be possible without financial remuneration for GPs.

“*The improvements come with if you had more time, if you were financially rewarded […] to set up a specific call-recall system where you would check in with people [but] it's hard to see how you can improve it as you are now.” (GP05)*

In addition to improved care for the patient with dementia, a structured care approach could positively impact carer burden.

“*I think from the point of view of the families of people with dementia it would probably be good in that they'd feel they're not left out to sea.” (GP12)*

Lack of time and resourcing in General Practice were highlighted as major barriers by all participants to dementia and other chronic diseases. Multiple GPs explained that the reason there has been little change in the contract model in primary care is due to disconnect between policy makers and clinicians.

“*You have people in offices making decisions but nobody on the ground making the decisions and like that's where it totally gets lost.” (GP12)*

#### GP Recruitment & Training

Participants explained that remuneration alone is not enough to improve the quality of future care. Recruitment and training of more physicians into General Practice is paramount.

“*Even if you gave me a lot of money to go to that meeting I still have 20 patients waiting here. We need more doctors. And doctors are expensive to train and there is a lag period of a decade.” (GP01)*

The need for a continued focus on education for current GPs was emphasized by participants. Multiple aspects of dementia care were identified which could be improved with further training and knowledge.

“*It would be really good if we could do something related to the legal side of dementia. I think that is an area that GPs struggle with, particularly because things are changing at the moment.” (GP02)*

“*If we're more empowered as GPs and knowledgeable, [if] we're more comfortable with the medication, we can prescribe while we're waiting.” (GP08)*

“*Not every GP realizes what the resources are and probably aren't using them to their full potential either.” (GP08)*

Generational gaps in knowledge and training of dementia were highlighted by one GP.

“*GPs of my generation haven't been formally taught about dementia. A lot of the current concepts and approaches weren't around when I was going through college, a lot of the knowledge about dementia.” (GP07)*

Another GP emphasized the need for training incentivization to formalize special interests within General Practice to improve access to specialist care and reduce the burden on secondary services.

“*If you had a GP, maybe 30 or 40 of us, who were really up-skilled. We call them GPSIs. If you are having a little bit of trouble you send out to them and they could see them in a week's time as opposed to a year's time in clinic […] And this is starting to happen. But […] it needs to be incentivized a little bit more with the training. You have a lot of people kind of doing what they like.” (GP01)*

### Theme 2: Addressing Disparities in Secondary Care

#### Accessing the Diagnosis & Specialist Support

The support of secondary care was deemed by participants to be necessary for the appropriate management of patients with dementia.

“*With dementia care we can't do it on our own […] We're going to need a psychogeriatrician when things go wrong. We need expertise. We're going to need a memory clinic to confirm the diagnosis and subtitle it so they're on the right treatment and the right path.” (GP08)*

The majority of participants highlighted difficulties accessing secondary care and/or the absence of particular services in their practice regions, which impacts time to diagnosis and access to expert supports.

“*The public waiting lists to see a geriatrician are very, very long so sometimes getting the diagnosis is difficult and time-consuming.” (GP02)*

Some GPs argued that if access to resources in primary care was enhanced the diagnosis of dementia could be finalized in primary care.

“*It would be great for GPs to have access to CT because most GPs would need a clear CT scan to make the diagnosis of dementia but wouldn't have access to CT in the hospitals.”*

Many GPs commented on the quality of services in public secondary care and were divided in their opinions.

“*We've got a really good geriatrician […] who is very open to phone calls and to discussion of patients and to accessing patients more rapidly […] They're sensitive and community-centered. It helps that you're not referring into a big, enormous hospital.” (GP09)*

“*If they're going publicly it's a lot harder to get them seen. They're waiting a lot longer and then it really depends on who they meet when they go in there the quality of the service they get.” (GP12)*

A few participants suggested for specialists to become more involved in the community and nursing homes.

“*The other place geriatricians could probably improve would be to come out to the nursing homes a little bit. Even once or twice a year for the few tricky ones.” (GP01)*

#### Acute Care Pathway

Participants highlighted the importance of advanced directives in minimizing inappropriate out-of-hours care.

“*They all need personalized care and they're quite unique in that you have to know them and what level of care they are due to receive whereas when it's an out-of-hours setting they can be treated by doctors who don't know them who can sometimes suggest treatments that they really shouldn't be for or referring them to secondary or tertiary centers that they probably shouldn't be going to.” (GP03)*

But the need for acute services was described as inevitable in certain instances. Multiple GPs suggested establishing a pathway for patients with dementia to access acute care safely and efficiently.

“*It would be nice to have a phone number or a person or team that you could contact and say, ‘Look, this person is coming in and they've got dementia. Can you fast-track them through [the Emergency Department]?’ That would be the ideal situation.” (GP02)*

One GP contended that a diagnosis of dementia impacts the quality of care in an acute care setting.

“*If you send an 80-year-old in who is fully compos mentis and she goes in with a pneumonia she'd somehow be treated differently to the 80-year-old who is sent in who has an MMSE of 22/30.” (GP12)*

#### Patient Expenses

Multiple participants highlighted funding as a barrier to patients and their carers accessing secondary services. They contended that patients with a formal diagnosis of dementia should be entitled full access to publicly funded health services.

“*A lot of patients over 70 have a GP visit card but there are not entitled to physiotherapy, they're not entitled to anything else bar coming to see us. So it can be hard to get them into the other services. You are asking them to pay for it all.” (GP04)*

### Theme 3: Community-Centered Care Equals Patient-Centered Care

GPs stressed that health outcomes and quality of life for patients with dementia are optimized by prioritizing community-centered care.

“*Where does the patient want to be? They want to be at home. They'd prefer to be cared for at home than in a nursing home. So get them the support, get them the help, and make sure they stay there as long as they can.” (GP09)*

They further emphasized the cost-effectiveness of community services to avoid unnecessary inpatient admissions or premature nursing home commitments.

“*When you balance maybe having an additional OT (occupational therapist) or speech and language therapist against a few days inpatient care after a crisis it would make good economic sense.” (GP03)*

However, GPs highlighted barriers to community-based care in Ireland.

“*A big void in community care is resource access. Most GPs […] know what sort of ancillary services you need for community-based dementia patients but what you need and what you have access to are usually not the same thing.” (GP03)*

Multiple ideas were proposed by GPs to improve community services for patients with dementia and their carers.

“*It would be great if there were dementia nurses in the community […] that would call to the house and could give [the family] an hour a week for three or four weeks and just go through everything to expect in the future, to go through planning ahead, to go through services available to them.” (GP04)*

“*I think we all need dementia cafes or dementia drop-in clinics […] So that there's somewhere safe you can go as a carer, where you'll get support and where the patient with dementia will fit in and not be frowned upon.” (GP08)*

One GP argued that community nurses and drop-in clinics are not enough and that sheltered accommodations need to become widely distributed.

“*We really miss the sheltered accommodation in Ireland […] People are often too well to move into a nursing home or […] they have really neglected themselves and then a crisis happens and they end up in a nursing home. It would be nice if there was more of a tiered system where there was a sheltered accommodation component in the middle.” (GP02)*

### Theme 4: Linking a Dementia Network

Most GPs did not attend multidisciplinary primary care team (PCT) meetings and did not have a local functioning PCT. A lack of coordination among community services was highlighted by many GPs as a challenge to providing care to patients with dementia.

“*It's not well-coordinated and there's no good communication amongst the different [services] […] That doesn't happen because there isn't a primary care team working together where in an ideal world you'd sit down and discuss the patient and see what needs doing, what needs to happen.” (GP08)*

In contrast to this, a minority of GPs were actively involved in their PCT and highlighted benefits to coordinated care teams.

“*Often times the primary care team might be the first place that somebody will air a concern about a patient. Perhaps they might be slipping a bit, we mightn't have noticed it.” (GP06)*

### Theme 5: Universal Access to Care

The services available to each GP participant varied by geography, highlighting a fragmented system of services in Ireland.

“*You have a memory clinic in one area, you have a geriatrician only in another area, you have a psychogeriatrician in another area. So I think that needs to be rationalized. Every area should really have access to the same secondary care.” (GP06)*

GPs emphasized the need for uniform access to care irrespective of geography.

“*It shouldn't be different in Kerry and Cork and Waterford and Tipperary. It should be a national roll out that every area has the same access to memory clinics, has the same access to community services, that it's all uniform and fair and balanced.” (GP08)*

### Theme 6: Raising Public Awareness

A few participants acknowledged an improvement in national public awareness of dementia. It was reported, however, that awareness is limited to milder forms of the disease.

“*A lot of patients who have dementia on the television are actually quite well […] But I think the difficulty for some dementia patients, obviously if you have more advanced dementia, it's a bit like the child throwing a tantrum in the supermarket.” (GP05)*

GPs proposed for future public awareness efforts to be focused on preventing crises in the community.

“*Everybody knows about cardiovascular awareness […] But people aren't aware of any cognitive preventative tools. I think increasing awareness there is pretty important because there are certain breeding grounds for cognitive deterioration and there are certain environments that are quite good at being a protective factor for cognitive deterioration.” (GP03)*

## Discussion

### Summary of Main Findings

Our findings reveal that GPs are challenged by dementia care because of a lack of time and funding to provide structured care. They have variable access to secondary care and inadequate community-based resources. Their expressed view is that dementia care in Ireland can be improved by establishing a structured care program in primary care, enhancing community resourcing, formalizing local dementia networks, and standardizing dementia resources nation-wide.

### Comparison With the Literature

Our findings echo previous studies reporting barriers to dementia care in General Practice, particularly on the issues of adequate time and resourcing (17–20). Time and remuneration as perceived barriers to care may be more notable in countries where the GPs are remunerated directly for the services that they provide, such as in the USA, Canada or Ireland (21). Studies have shown that remuneration for GPs and structured care approaches lead to improvements in quality of care (22–25). A pay-for-performance scheme introduced in England financially remunerates GPs for achieving standards set out in the Quality and Outcomes Framework for chronic disease management, including dementia care. Implementation of the scheme has been shown to help consolidate evidence-based methods, improve the quality of incentivized aspects of care, and encourage greater consistency of care irrespective of deprivation (22, 23). Structured care models remunerate GPs for the management of a given disease, establishing formal requirements for registering, recording, and documenting care processes. Care models have been established for a few chronic diseases in primary care in Ireland, including diabetes (26). Although preliminary results of the national diabetes “cycle of care” program have not been published, regional structured diabetes care programs in Ireland have demonstrated improvements in the quality of care delivered to patients with type 2 diabetes mellitus over time (24, 25). All GP participants advocated the need for remuneration and structured dementia care in General Practice given the complexity and time-intensive nature of the disease. The exclusion of dementia from the list of chronic diseases to receive national funding for structured care programs in Ireland highlights the lack of prioritization of dementia and ongoing barriers to quality care.

The efficacy of coordinated care teams to support managing dementia in primary care has been evaluated through few clinical trials with mixed results. A randomized controlled trial in the United States found that one year of care management by an interdisciplinary team led by an advanced practice nurse working with the patient's carer and integrated with primary care resulted in significant improvement in the quality of care and behavioral and psychological symptoms of dementia among patients and carers (27). Such evidence supports funding coordinated care programs for dementia. It also suggests that effective primary care teams (PCT), defined as multidisciplinary teams of community-based allied health care professionals and GPs, may hugely benefit this patient cohort. However, other randomized trials to evaluate the effectiveness of case management among older adults with early symptoms of dementia and their primary informal caregivers have not been as convincing (28, 29). One trial found that a 12-month, nurse-led, case management intervention directed at patients with abnormal screening for symptoms of dementia and their caregivers offered no benefits over usual care with respect to quality of life of the patient and caregiver; however, poor intervention fidelity was reported (28). As well, the findings of a subsequent trial to evaluate the effectiveness of case finding by allied health teams to improve dementia diagnosis rates and quality of care were not significant (29). This may suggest that coordinated care interventions offer more benefit to patients with dementia and their carers after a formal diagnosis of dementia has been made.

Our study highlights current challenges in Ireland with respect to coordinated, integrated care in General Practice. A PCT system was adopted in Ireland in 2001 to provide patients with “one-stop-shop” access to a range of health care providers (30); however, the delivery of PCTs has been suboptimal. A study by the Irish College of General Practitioners in 2011 found that just over half of GPs were part of a PCT and the majority felt the team functioned poorly (31). In our study, only a fraction of the GPs participated in PCT meetings and felt their local PCTs functioned effectively, while the great majority of GPs did not. In contrast, the uptake of multidisciplinary teams outside of Ireland has seen varying degrees of success in primary care (32). Unfortunately, the dysfunction and fragmentation of PCTs in Ireland negatively impacts the quality of dementia care in General Practice as access to allied health services is poorly coordinated and GPs are left isolated and limited in their ability to provide care.

In contrast with previous studies, the challenges of disclosing the diagnosis of dementia did not emerge as a major theme in this study. Research suggests the majority of people with or without cognitive impairment prefer to be informed about a diagnosis of dementia to maintain autonomy and carry out advanced care planning (33). GPs have reported challenges in making and disclosing the diagnosis of dementia to patients, which has led to the development of educational resources and training for GPs in Ireland around this topic (9). Participants in this study had recently completed a dementia educational program, which may explain why this theme did not emerge.

To our knowledge, this is the first qualitative study in Ireland to gather GPs' perspectives on improving the quality of dementia care in General Practice. As central players in the care for patients with dementia these perspectives are crucial for guiding future quality improvement efforts for dementia care in Ireland. Further, GPs' opinions on auditing dementia care have not previously been evaluated. Many GPs noted that the greatest benefit of performing a dementia-focused audit was developing a dementia register for their practice as part of the process. GPs explained that the accurate coding of patients with dementia facilitated care provided by all members of their practice team, including administrators, nurses and GPs. The feasibility of establishing a National Dementia Registry for Ireland has been considered (34). There is general agreement in the literature that disease registries can facilitate improvements in policy, patient care and research efforts, and have a role to play in national public health strategies (34). Our study findings suggest that a dementia-specific registry improves care delivered by GPs to patients with dementia, which offers new evidence to further support the development of structured dementia care in Ireland.

### Strengths & Limitations

This study used a qualitative design, encouraging participants to express detailed opinions on the complex topic of dementia. The study population was varied by age, sex, practice size and setting, and nursing home commitments, which enriched the data set. All interviews were analyzed independently by two researchers over multiple assessments to ensure the themes accurately represented the data set.

Regarding study limitations, we acknowledge that the GPs that participated in the study may be considered experts in caring for dementia, possibly introducing bias in study findings. However, it was paramount that opinions were collected from GPs with strong clinical experience and an interest in dementia care. They also had experience performing a dementia-focused audit, which allowed us to gather feedback on aspects of a structured care approach.

### Future Implications

By emphasizing the need for structured care and taking a chronic disease management approach, the findings of this study may inform future policy-making, funding and care models, and research efforts to improve the quality of dementia care in Irish General Practice.

## Conclusion

While the INDS recognized the central role played by GPs in comprehensive care, dementia care in Irish General Practice is currently time-limited and under-resourced. Structured care models must be expanded to cover more chronic diseases in General Practice, including dementia care. Multidisciplinary PCTs and community-based dementia care services should be further developed and standardized nationally in order to ensure consistent optimal quality care across the country.

## Data Availability Statement

The raw data supporting the conclusions of this article will be made available by the authors, without undue reservation.

## Ethics Statement

Ethical approval was granted by the Clinical Research Ethics Committee of the Cork Teaching Hospitals. The participants provided their written informed consent to participate in this study.

## Author Contributions

TF conceived, designed, and obtained funding for this study. MB obtained ethical approval for the study and conducted and transcribed each interview. MB and TF made substantial contributions to the data analysis process and the writing of successive drafts of the manuscript. Both authors have read and approved the final draft.

## Conflict of Interest

The authors declare that the research was conducted in the absence of any commercial or financial relationships that could be construed as a potential conflict of interest.
