# Supplementary material for: Improving the Quality of Dementia Care in General Practice: A Qualitative Study
Source: Front Med (Lausanne). 2020 Nov 25;7:600586. doi: 10.3389/fmed.2020.600586 (PMC7724029; doi:10.3389/fmed.2020.600586)
Supplement: Supplementary file 2 [file Data_Sheet_2.PDF]

Improving the Quality of Dementia Care in General Practice: A Qualitative Study

Participant Details

STUDY TITLE: Improving the Quality of Dementia Care in General Practice: A Qualitative Study

NAME OF CHIEF INVESTIGATOR: Dr. Tony Foley

NAME OF MEDICAL STUDENT: Meghan Bourque

The purpose of the following questions is to gather demographic details to compliment the qualitative data gathered in today's interview. Please identify if there are any concerns with the details requested below.

Participant Name: ___________________________________

1. Please circle your identified sex: **MALE** **FEMALE** **OTHER**

2. Please circle the range that corresponds to your current age in years:

   20-29 **30-39** **40-49** **50-59** **60-69** **70-79** **80-89**

3. How many years have you been in practice as a GP? _________________

4. How would you describe the location of your current practice? **URBAN** **RURAL** **MIXED**

5. What is the estimated number of total patients in your practice? _________________

6. What is the estimated number of patients with dementia in your practice? _________________

7. Do you have nursing home commitments? **YES** **NO**
