# Supplementary material for: Improving the Quality of Dementia Care in General Practice: A Qualitative Study
Source: Front Med (Lausanne). 2020 Nov 25;7:600586. doi: 10.3389/fmed.2020.600586 (PMC7724029; doi:10.3389/fmed.2020.600586)
Supplement: Supplementary file 3 [file Data_Sheet_3.PDF]

INTERVIEW GUIDE

STUDY TITLE: Improving the Quality of Dementia Care in General Practice: A Qualitative Study

NAME OF CHIEF INVESTIGATOR: Dr. Tony Foley

NAME OF MEDICAL STUDENT: Meghan Bourque

PART 1 – Practice Details

1. Can you tell me a bit about yourself and your practice?
2. Can you tell me about dementia care in your practice?

PART 2 – Opinions on the Quality of Dementia Care

3. What aspects of dementia care work well in your practice?
4. Why do these aspects work well ie. what are the major facilitators to care?
5. What aspects of dementia care do not work well?
6. Why do these aspects of care not work well ie. what are the major barriers to care?
7. How would you rank the quality of dementia care in your own practice?
8. How do you think you can improve the quality of dementia care in your own practice?
9. Can you describe any initiatives (other than audit) that you have undertaken to improve the quality of dementia care in General Practice?
10. Could you tell me about your views of dementia care on a national level?

PART 3 – Auditing Dementia Care

11. Can you describe your experience of performing an audit – specifically of auditing your dementia care?
