# Supplementary figures and images for: Improving the Quality of Dementia Care in General Practice: A Qualitative Study
Source: Front Med (Lausanne). 2020 Nov 25;7:600586. doi: 10.3389/fmed.2020.600586 (PMC7724029; doi:10.3389/fmed.2020.600586)

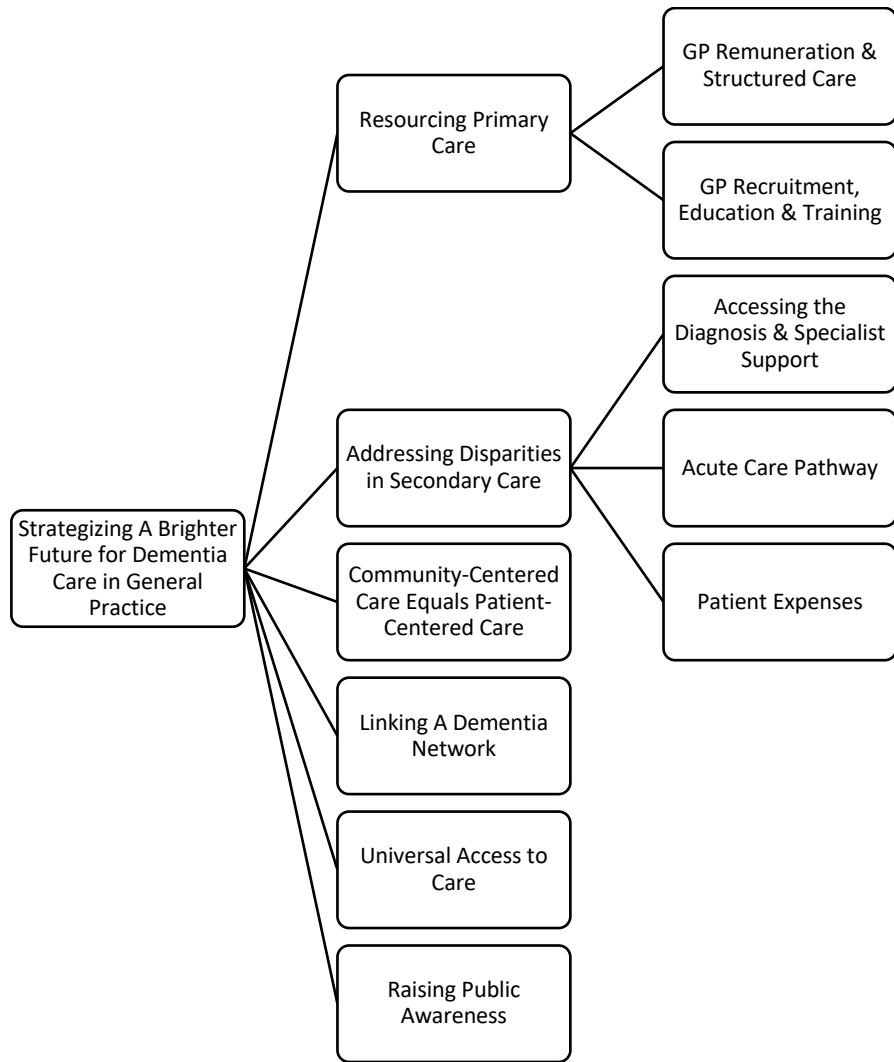

Supplement: Supplementary file 4 [file Data_Sheet_4.PDF]

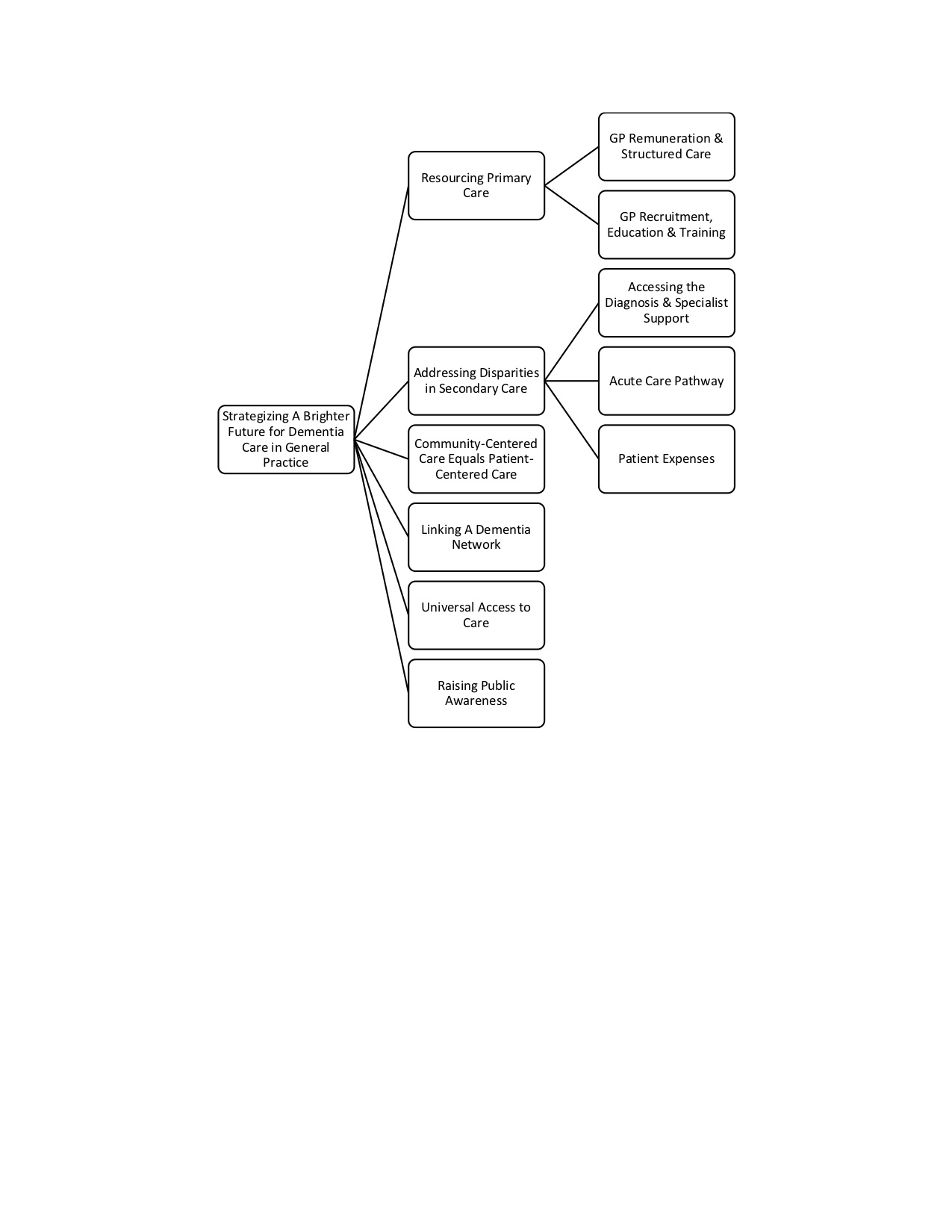

Supplement: Supplementary file 5 [file Image_1.JPEG]
