# Supplementary material for: Improving the Quality of Dementia Care in General Practice: A Qualitative Study
Source: Front Med (Lausanne). 2020 Nov 25;7:600586. doi: 10.3389/fmed.2020.600586 (PMC7724029; doi:10.3389/fmed.2020.600586)
Supplement: Supplementary file 1 [file Data_Sheet_1.PDF]

Improving the Quality of Dementia Care in General Practice: A Qualitative Study

Invitation to Participate

Dear Dr (insert name),

I am writing to invite you to participate in a one-to-one research interview to explore your views on improving the quality of dementia care in General Practice. You have been invited to participate because you are a practicing GP and/or have completed the Dementia in Primary Care module at UCC.

There are three major aims of the study: 1) To gain GPs' perspectives on improving the quality of dementia care in General Practice; 2) To gain GPs' perspectives on performing a dementia-focused audit; and 3) To gather feedback on audit resources and tools.

If you decide to participate, the research interview will be scheduled at a time and place that is convenient for you. A member of the research team will ask questions to explore the major aims outlined above and you will be have the option to share your audit report and data. The interview will be audio-recorded and is estimated to take 20-30 minutes to complete.

An information leaflet is enclosed that provides further detail about the project. We ask that you please complete the form on the following page and return it in the pre-stamped envelope provided. If you agree to participate in the study you will be contacted by a member of the research team promptly.

Your participation is very much appreciated and will contribute to national efforts to improve the quality of dementia care in General Practice.

Yours Sincerely,

Meghan Bourque

Meghan Bourque

Improving the Quality of Dementia Care in General Practice: the GP Perspective

Participation Form

Please complete this form and return to us in the envelope enclosed.

Name: ____

Contact number: ___________________________________

☐ Yes, I would like to participate in a one-to-one interview to share my perspectives on improving the quality of dementia care in General Practice.

☐ No, I do not want to participate in a one-to-one interview to share my perspectives on improving the quality of dementia care in General Practice.

☐ I would like to hear more information before making a decision to participate or not.

If you have selected 'No' we would appreciate any feedback on why you have chosen not to participate: (ex. time constraints, lack of interest)

____

____

____

____

Signature: ____ Date: ____________________

Improving the Quality of Dementia Care in General Practice: A Qualitative Study

Letter of Information

**STUDY TITLE**:	Improving the Quality of Dementia Care in General Practice: A Qualitative Study
**NAME OF CHIEF INVESTIGATOR:**	Dr. Tony Foley
**STUDENT NAME:**	Meghan Bourque

The purpose of this letter is to provide information on relevant questions about the study. Please read each section carefully and if you have any further questions, do not hesitate to contact a member of the research team.

WHY IS THIS RESEARCH BEING DONE?
In 2014, the Irish National Dementia Strategy was established to improve the quality of care for people living with dementia. As part of the implementation of the strategy, the PREPARED project was launched and is being led by the Department of General Practice at UCC. In 2016-17, the PREPARED project developed resources (audit tools and guide) to support GPs choosing to audit the care of people with dementia, as well as a Dementia in Primary Care (DIPC) module. As part of the module, GPs are educated on the audit resources and encouraged to use them to perform a mandatory dementia-focused audit. The module is now in its second iteration and the PREPARED project is looking for feedback from participants on their audit experiences. At the same time, we would like to gather GPs' opinions on how to continue to improve the quality of dementia care in Ireland. This feedback will be used to inform national bodies on the benefits and challenges of auditing dementia care in General Practice, as well as guide future quality improvement efforts for dementia care.

PURPOSE OF THE STUDY
The purpose of the study is to gain GPs' perspectives on improving the quality of dementia care in General Practice and performing dementia-focused audits. The study also aims to gather feedback on resources developed to assist GPs in performing dementia-focused audits.

STUDY PROCEDURE
Participation in the study will involve a one-to-one interview with a member of the research team to explore perspectives on improving the quality of dementia care in General Practice, hear about GP' experiences performing a dementia-focused audit, and collect feedback on audit resources. You will also have the option to share your audit report and data. If you decide to participate, the interview will be scheduled at a time and place that is convenient for you. It will be audio-recorded and is estimated to take 20-30 minutes to complete.

CONFIDENTIALITY
All information obtained during the study will be strictly confidential. Your identity and all identifiable information will be removed from survey data and only investigators named on this form will have access to the data. Data will only be used for the purpose of this study and will be securely held for a minimum period of ten years after completion of the research project.

WHAT WILL HAPPEN WITH THE RESULTS OF THIS STUDY?
The findings of this study will be written up in a report for the PREPARED team and may be presented at meetings, conferences and in other publications. In all cases, only anonymous extracts or quotes will be reported. A copy of the findings will be made available to you upon your request.

POTENTIAL RISKS
Participation in the study is unlikely to be associated with any harm or discomfort. If you feel uncomfortable with any aspect(s) of the interview, you may choose to end the interview immediately and withdraw the inclusion of your data in the study.

POTENTIAL BENEFITS
Participants may be able to record the interview activity as a personal/research CPD credit. As well, study findings may be used to continue to improve the quality of dementia care in Ireland and the resources available to GPs for continued quality improvement in their practices.

WITHDRAWING FROM THE STUDY
You can withdraw from the study and any data you have contributed will be removed up until 1 week after you complete the interview.

PAYMENT OR REIMBURSEMENT
Participation in the study is voluntary and you will not be paid.

WHO HAS REVIEWED THIS STUDY?
This research project gained approval from the Clinical Research Ethics Committee (CREC) at UCC. If you decide to take part, you will be asked to sign a consent form. You are free to withdraw at any time. If you have any concerns after the interview, please do not hesitate to contact the research team, as detailed at the top of this leaflet.
